# MARSBOx: Fungal and Bacterial Endurance From a Balloon-Flown Analog Mission in the Stratosphere

**DOI:** 10.3389/fmicb.2021.601713

**Published:** 2021-02-22

**Authors:** Marta Cortesão, Katharina Siems, Stella Koch, Kristina Beblo-Vranesevic, Elke Rabbow, Thomas Berger, Michael Lane, Leandro James, Prital Johnson, Samantha M. Waters, Sonali D. Verma, David J. Smith, Ralf Moeller

**Affiliations:** ^1^Aerospace Microbiology Research Group, Department of Radiation Biology, Institute of Aerospace Medicine, German Aerospace Center, Cologne, Germany; ^2^Astrobiology Research Group, Department of Radiation Biology, Institute of Aerospace Medicine, German Aerospace Center, Cologne, Germany; ^3^Biophysics Research Group, Department of Radiation Biology, Institute of Aerospace Medicine, German Aerospace Center, Cologne, Germany; ^4^NASA Kennedy Space Center, Engineering Directorate, Kennedy Space Center, Merritt Island, FL, United States; ^5^Universities Space Research Association, Moffett Field, CA, United States; ^6^NASA Ames Research Center, Space Biosciences Research Branch, Moffett Field, CA, United States; ^7^Blue Marble Space Institute of Science, Moffett Field, CA, United States

**Keywords:** Mars simulation, fungal spores, spore survival, space, radiation, UV, balloon flight, stress resistance

## Abstract

Whether terrestrial life can withstand the martian environment is of paramount interest for planetary protection measures and space exploration. To understand microbial survival potential in Mars-like conditions, several fungal and bacterial samples were launched in September 2019 on a large NASA scientific balloon flight to the middle stratosphere (∼38 km altitude) where radiation levels resembled values at the equatorial Mars surface. Fungal spores of *Aspergillus niger* and bacterial cells of *Salinisphaera shabanensis*, *Staphylococcus capitis* subsp. *capitis*, and *Buttiauxella* sp. MASE-IM-9 were launched inside the MARSBOx (Microbes in Atmosphere for Radiation, Survival, and Biological Outcomes Experiment) payload filled with an artificial martian atmosphere and pressure throughout the mission profile. The dried microorganisms were either exposed to full UV-VIS radiation (UV dose = 1148 kJ m^−2^) or were shielded from radiation. After the 5-h stratospheric exposure, samples were assayed for survival and metabolic changes. Spores from the fungus *A. niger* and cells from the Gram-(–) bacterium *S. shabanensis* were the most resistant with a 2- and 4-log reduction, respectively. Exposed *Buttiauxella* sp. MASE-IM-9 was completely inactivated (both with and without UV exposure) and *S. capitis* subsp. *capitis* only survived the UV shielded experimental condition (3-log reduction). Our results underscore a wide variation in survival phenotypes of spacecraft associated microorganisms and support the hypothesis that pigmented fungi may be resistant to the martian surface if inadvertently delivered by spacecraft missions.

## Introduction

Mariner IV was the first successful robotic mission to Mars producing surface photos and preliminary data used to model atmospheric pressure, layer heights, and temperature (Leighton et al., 1965; Binder, 1966; Fjeldbo and Eshleman, 1968). In the half century since that first pioneering mission, orbital and surface rover missions have continued to characterize the Mars environment – generally regarded as hostile to terrestrial life as we know it. The martian surface features highly desiccating conditions as well as extremely low pressure and temperature. Moreover, lacking a substantial atmosphere and with a weak magnetosphere (Acuña et al., 2001), non-ionizing UV radiation (100–400 nm) as well as high energy solar ionizing radiation (X-rays, Gamma rays, etc.) and galactic cosmic rays (GCR) bombard the planet’s surface (Kuhn and Atreya, 1979; Simonsen et al., 1990; Saganti et al., 2004; Hellweg and Baumstark-Khan, 2007; Barlow, 2008; Catling, 2009; Hassler et al., 2014; Martínez et al., 2017). With such an extreme radiation environment, from a terrestrial standpoint, the martian surface appears to be biocidal.

To ascertain where life can survive beyond Earth, experiments in the fields of space biology and astrobiology have examined the responses of terrestrial model organisms to simulated and real space conditions (Rothschild and Mancinelli, 2001; Moissl-Eichinger et al., 2016; DasSarma et al., 2020). Understanding microbial adaptations to either isolated and combined extreme environmental stressors helps (i) establish the limits of life on Earth as we know it; (ii) determine whether terrestrial life could survive on Mars; and (iii) refine the search for life in other extraterrestrial habitats (Horneck et al., 2010; Cockell et al., 2016). With the possible exception of the Viking missions, Mars has been unavailable to date for conducting controlled biological experiments; thus, extreme terrestrial analog environments have been widely used to test instrumentation and microbial survival outcomes (Marlow et al., 2008; Fairén et al., 2010; Suedfeld, 2010; West et al., 2010). Typically, martian analog environments are located on Earth’s surface in regions where aridity, temperature extremes, and elevated radiation dominate the landscape. For instance, the McMurdo Dry Valleys in continental Antarctica and high-elevation deserts in Australia and South America are frequently visited, analog destinations (Clarke and Persaud, 2004; Fletcher et al., 2012; López-Lozano et al., 2012; Heldmann et al., 2013; Musilova et al., 2015). However, high above Earth’s surface in the stratosphere (∼15–50 km), another Mars analog environment exists, presenting a unique combination of environmental insults that more closely resemble conditions on the Red Planet. In the middle stratosphere during daytime hours, the following Mars-like factors are simultaneously present: intense, full spectrum ultraviolet (UV) radiation, high energy ionizing radiation (including secondary scattering), desiccation, hypoxia, and ultralow temperatures and pressures (Clark and McCoy, 1965; Potemra and Zmuda, 1970; Vampola and Gorney, 1983; Keating et al., 1987; Clancy and Muhleman, 1993; Von Engeln et al., 1998; Seele and Hartogh, 1999; Shepherd, 2000; Lambert et al., 2007; Mertens et al., 2016; Caro et al., 2019). Taken together, these combined conditions cannot be found naturally anywhere on the surface of the Earth and would be challenging to easily reproduce in laboratory-based experiments.

Reaching the middle stratosphere is relatively achievable compared to suborbital and orbital spaceflight investigations. High-altitude scientific balloons have been used for more than eight decades to study the atmosphere and atmospheric phenomena (Winckler et al., 1959; Winckler, 1960; Murcray et al., 1969; Mertens et al., 2016; Caro et al., 2019) and more recently for conducting biological exposure experiments (Stevens, 1936; Simons, 1954; Sullivan and Smith, 1960; Rainwater and Smith, 2004; Beck-Winchatz and Bramble, 2014; Coleman and Mitchell, 2014; Smith et al., 2014; Khodadad et al., 2017; Smith and Sowa, 2017; Pulschen et al., 2018). In this study, we take advantage of a large scientific balloon mission to the middle stratosphere (∼38 km altitude) for exposing microorganisms and measuring their survival and metabolic responses while monitoring ionizing radiation levels and other pertinent environmental conditions. Four microorganisms relevant to astrobiology and space biology were flown inside the Microbes in Atmosphere for Radiation, Survival, and Biological Outcomes Experiment (MARSBOx) payload. The two bacterial extremophiles, *Salinisphaera shabanensis* and *Buttiauxella* sp. MASE-IM-9, were included to test the hypothesis that terrestrial microbial strains, isolated from extreme Mars-analog environments, can withstand the stress factors of a martian-like environment. The fungus *Aspergillus niger* and the bacterium *Staphylococcus capitis* subsp. *capitis* were included in this study because they are human-associated and opportunistic pathogens, and have both been previously detected on the International Space Station (ISS). Thus, they are likely to travel to Mars in crewed space missions (Novikova et al., 2006; Checinska et al., 2015; Be et al., 2017; Mora et al., 2019; Sobisch et al., 2019). Moreover, spores from *A. niger* might resist space travel on the outside of a spacecraft; therefore, understanding their survival potential in a Mars-like environment is of interest to planetary protection.

The MARSBOx design was a balloon-compatible, NASA-adapted version of hardware from the European Space Agency’s (ESA) biological exposure missions EXPOSE-E and EXPOSE-R aboard the ISS (Rabbow et al., 2009; Rabbow et al., 2012, 2015), using the transport and exposure box (Trex-Box) from the European MASE project (Beblo-Vranesevic et al., 2017a). To adjust for Mars atmospheric conditions, the Trex-Box was filled with a Mars gas mixture at 5–10 mbar during the mission. Herein, we report results from the first MARSBOx mission and summarize environmental conditions that collectively indicate the most robust Mars analog.

## Materials and Methods

### Test Organisms, Media, and Sample Preparation for Flight

A summary of the microorganisms used in this study can be found in Table 1. *Aspergillus niger* (N402) spores were harvested after 3 days of incubation at 30°C from complete medium agar plates [CM; composition: 55 mM glucose, 11 mM KH_2_PO_4_, 7 mM KCl, 178 nM H_3_BO_3_, 2 mM MgSO_4_, 76 nM ZnSO_4_, 70 mM NaNO_3_, 6.2 nM Na_2_MoO_4_, 18 nM FeSO_4_, 7.1 nM CoCl_2_, 6.4 nM CuSO_4_, 25 nM MnCl_2_, 174 nM EDTA; 0.5% (w v^–1^) yeast extract and 0.1% (w v^–1^) casamino acids, 15 g agar per Liter] by flooding the plates with sterile, saline solution (0.9% NaCl) and gently scraping the spores out using a cotton stick.

**TABLE 1:** Test microorganisms used in this study.

Organism	Classification	Growth	Tested as	References
***Aspergillus niger* N402**	Filamentous fungus (mold)	Minimal Medium 30°C	Spore monolayer (10^7^ spores mL^–1^) or spore multilayer (10^8^ spores mL^–1^) desiccated in water	Bos et al., 1988
***Staphylococus capitis* subsp. *capitis* K1-2-2-23**	Gram-(+) bacteria	Tryptic Soy Broth (TSB) or Agar (TSA) 37 °C	Cell multilayer (1 × 10^9^ CFU mL ^–1^) desiccated in PBS	Sobisch et al., 2019
***Salinisphaera shabanensis***	Gram-(–) bacteria Halophilic	Marine broth or agar 30°C	Cell multilayer (2 × 10^8^ CFU mL^–1^) desiccated in PBS (10% NaCl) or medium.	Antunes et al., 2003
***Buttiauxella* sp. MASE-IM-9**	Gram-(–) bacteria	Tryptic Soy Broth (TSB) or Agar (TSA) 30°C	Cell multilayer (2 × 10^8^ CFU mL^–1^) desiccated in PBS or medium.	Cockell et al., 2018

**FIGURE 1:**
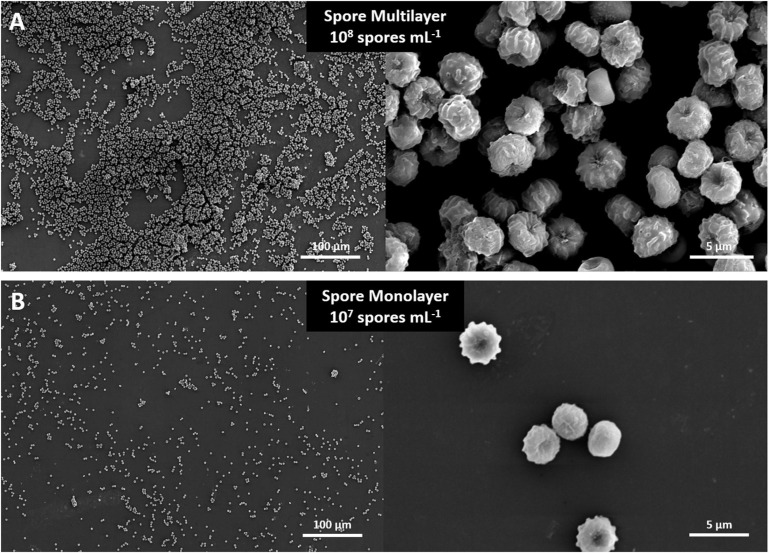
SEM images of *Aspergillus niger* spores in **(A)** multilayer (10^8^ spores mL^–1^) and **(B)** monolayer (10^7^ spores mL^–1^).

The spore suspensions were then filtered through a sterile filter with 22–25 μm pore size (Miracloth) to remove hyphal fragments and kept at 4°C. Titer determination was done using an improved Neubauer cell count chamber on vortexed suspensions; 20 μL of spores were then spotted onto round quartz disks (6 mm ∅, 1 mm thickness; MolTech) in triplicate and left to dry at room temperature (22°C) on the bench. Two spore concentrations were prepared for the MARSBOx flight: 1 × 10^7^ spores mL^–1^ (spore monolayer) and 1 × 10^8^ spores mL^–1^ (spore multilayer). Presence of spore multilayer and monolayer was determined qualitatively with a scanning electron microscope (JSM-6510, Jeol), operated at 10 Kv (Figure 1). Further experiments using agar plates were done with minimal medium (composition: 55 mM glucose, 11 mM KH2PO_4_, 7 mM KCl, 178 nM H_3_BO_3_, 2 mM MgSO_4_, 76 nM ZnSO_4_, 70 mM NaNO_3_, 6.2 nM Na_2_MoO4, 18 nM FeSO_4_, 7.1 nM CoCl_2_, 6.4 nM CuSO_4_, 25 nM MnCl_2_, 174 nM EDTA, 15 g agar, per Liter).

*Staphylococcus capitis* subsp. *capitis* strain K1-2-2-23 (DSM 111179) was cultivated in tryptic soy broth (TSB; BD Difco; composition: tryptone 17.0 g, soy peptone 3.0 g, glucose 2.5 g, NaCl 5.0 g, K_2_HPO_4_ 2.5 g, per Liter) at 37°C under constant agitation at 200 rpm for 18 h. Stationary phase cells were harvested by centrifugation (4000 rpm) in 40 mL culture for 5 min. Cells were washed by removal of the supernatant, resuspension of the pellet in 40 mL phosphate buffered saline (PBS; composition: Na_2_HPO_4_ 7.0 g, KH_2_PO_4_ 3.0 g, NaCl 4.0 g, per Liter, pH 7.5), and centrifugation for 5 min at 4000 rpm. The washing step was repeated once and after the last centrifugation step, the pellet was resuspended in 10 mL PBS. Thirty μL of this concentrated cell suspension were pipetted onto the quartz disks and left to dry at room temperature (22°C) on the bench. The absolute number of cells per quartz disk was determined by standard plate counts on tryptic soy agar (TSA; composition: tryptone 17.0 g, soy peptone 3.0 g, glucose 2.5 g, NaCl 5.0 g, K_2_HPO_4_ 2.5 g, agar 15 g, per Liter) to be 1.4 × 10^9^ cells per disk, resulting in a multilayer of bacterial cells.

The facultative anaerobes, *Salinisphaera shabanensis* and *Buttiauxella* sp. MASE-IM-9, were cultivated in liquid microoxic Marine Broth (BD Difco; composition: peptone 5.0 g, yeast extract 1.0 g ferric citrate 0.1 g, NaCl 19.45 g, MgCl_2_ 5.9 g, MgSO_4_ 3.24 g, CaCl_2_ 1.8 g, KCl 0.55 g, NaHCO_3_ 0.16 g, KBr, 0.08 g, SrCl_2_ 34.0 mg, boric acid 22.0 mg, Na_4_SiO_4_ 4.0 mg, NaF 2.4 mg, NH_4_NO_3_ 1.6 mg, and Na_2_HPO_4_ 8.0 mg, per Liter) and microoxic TSB, respectively, in sterile serum bottles with headspace pressure and gas composition of ∼1,010 mbar and N_2_/CO_2_ (80/20 v/v), respectively. Cell concentration was determined via cell counting in a Thoma counting chamber. Stationary phase cells from an overnight culture of each strain were harvested by centrifugation (14,500 *g*) of 1 mL culture for 15 min. To assess possible differences in survival between cells dried in medium (UV absorption of medium components) or dried in non-absorbing buffer, after the centrifugation step either (i) 950 μL of the supernatant were removed, cells were resuspended in the remaining growth media, and 50 μL were applied on each quartz disk; or (ii) cells were washed with 1 mL PBS (for *S. shabanensis* the NaCl content was adjusted to 10%), again centrifuged for 15 min at 14,100 *g*, the supernatant (950 μL) was removed, and 50 μL were applied on each quartz disk. The desiccation process was conducted under oxic conditions on the bench. The absolute number of cells per quartz disk was determined by standard plate counts on Marine agar/TSA to be ∼10^8^ cells per disk for both strains.

### Balloon Payload

**FIGURE 2:**
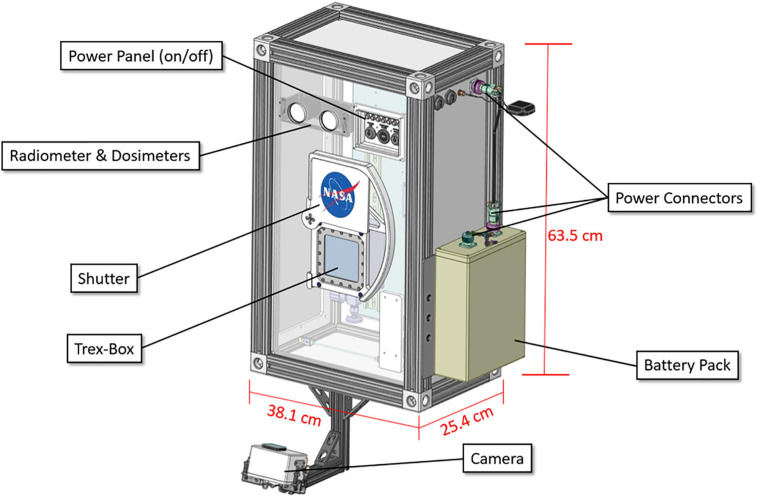
MARSBOx payload labeled model.

**FIGURE 3:**
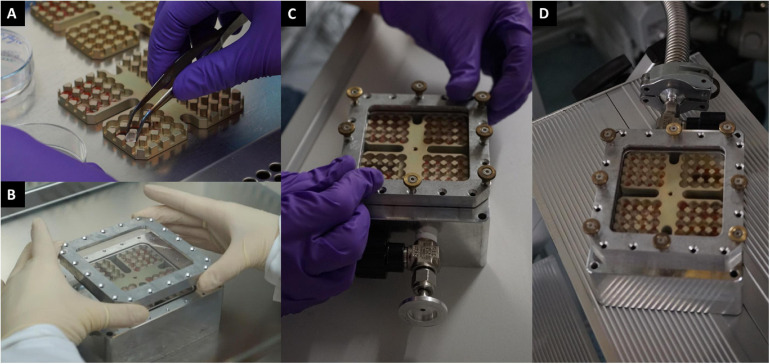
Trex-Box sample preparation. Dimensions of the Trex-Box are 13.5 cm × 13.5 cm × 5.0 cm. The container is a gastight closable stainless-steel box with one borehole which allows an exchange of internal atmosphere. **(A)** Quartz disks harboring the microbial samples being placed onto the Trex-Box; **(B)** covering the Trex-Box with a suprasil glass that allows for full UV-VIS exposure; **(C)** screws were used to tighten and seal the container; **(D)** Earth’s atmosphere being replaced with Mars-gas mixture.

**FIGURE 4:**
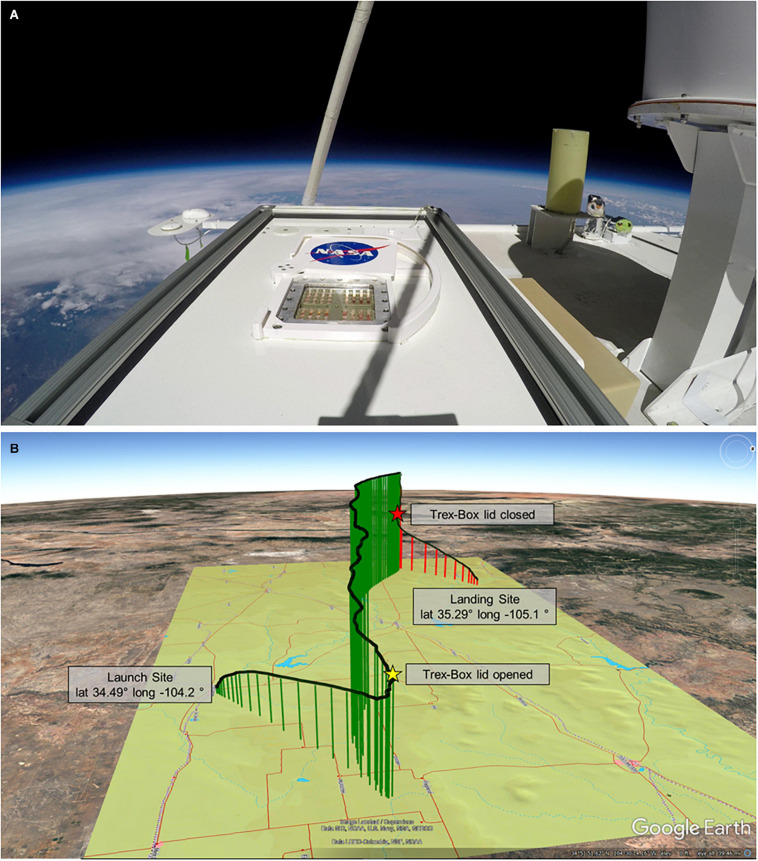
Balloon flight **(A)** Image from atop the MARSBOx payload and Trex-Box in the stratosphere during the flight. **(B)** Balloon flight path; stars mark opening and closing of the Trex-Box lid, which corresponds to UV-VIS radiation exposure beginning and ending.

The MARSBOx payload (38.1 cm × 25.4 cm × 63.5 cm; mass 18 kg) was built for simple mounting and integration onto the exterior of large scientific balloon gondolas (Figure 2). Biological samples were enclosed within a pressurized, shielded container (Trex-Box) (Beblo-Vranesevic et al., 2017a) with a rotatable shutter that prevented solar radiation exposure during ascent and descent (i.e., experimental initiation/termination). Covering the Trex-Box was suprasil glass: 8 mm thick, with a long pass cut off of ∼170 nm (with 0% transmission), and magnesium fluoride (MgF_2_), with a long pass cut off of ∼110 nm (custom made by MolTech, Germany) (Figure 3B). During the balloon flight, the MARSBOx system controlled the exposure to UV radiation so that the samples were only exposed at stratosphere altitudes [Figure 4 and Supplementary Videos 1 (ascent), **2** (descent)]. Motors, gears, and the shutter were held together by a frame composed of aluminum cutouts and 3D-printed polycarbonate-ABS components. T-slotted 80/20 aluminum extrusions formed the framework of the payload, with detachable, white powder-coated aluminum panels on each face of the MARSBOx. Angle brackets on the back plate were used to mount the system onto the balloon gondola. The front panel data port contained one micro-USB port, six light emitting diodes (LEDs), and two key switches. One key switch was used to power on the system and the other was used to manually rotate the shutter lid (for loading and removing Trex-Box with samples). The LEDs were programmed to indicate the state of the onboard computer’s health (OSD3358, Octavo Systems) and the status of the computer’s state machine, GPS receiver (GPS_FGPMMOPA6H, Adafruit Industries), camera system (Hero4 Black, GOPRO with Dash controller, CamDo), heater system, and the M-42 radiation dosimeter (Berger et al., 2019).

Other major payload components included three pressure sensors (MS5803, MEAS Switzerland; AMS5812, Analog Microelectronics; and BMP085, Bosch Sensortec), four temperature sensors, and a 9-axis Inertial Measurement Unit (IMU). While not flown on this mission, MARSBOx can carry additional instruments (e.g., UV radiometers) located behind the front panel of the payload. For the LDB #697NT flight, power was provided by a 14.8v 25.2 Ah lithium-ion polymer battery (CU-J141, BatterySpace) fastened in place with an ultem 3-D printed battery holder. The MARSBOx payload can also utilize a direct connection to the balloon gondola power source with an acceptable input range of 9V – 36V.

The design and technical details of the Trex-Box were previously described in detail (Beblo-Vranesevic et al., 2017a). Briefly, the design of the aluminum box was inspired by the EXPOSE mission series on the ISS (Rabbow et al., 2012; Rabbow et al., 2015) using a Trex-Box to control the transport of microorganisms during experiments (Beblo-Vranesevic et al., 2017a, 2020). The Trex-Box can be filled with gas and sealed, allowing for a sustained martian gas composition of 0.17% O_2_, 95% CO_2_, 0.07% CO, 2.6% N_2_, and 1.9% Ar during the course of the experiment at Mars-like pressures (∼7 mbar) (Figures 3C,D). The Trex-Box enabled testing of four different microorganisms without cross-contamination (an issue reported in past balloon experiments; see Díez et al., 2020) because each organism was set in one of the four quarter sections of each layer (Figure 3A).

### Stratospheric Flight Experiment

The Trex-Box consisted of two layers of an aluminum 64-well sample carrier, each holding quartz disks with microbial samples (see section “Test organisms, media, and sample preparation for flight”) that were either exposed to direct stratospheric UV radiation (UV-exposed, top layer) or shielded from UV (UV-shielded, bottom layer) (Table 2). The quartz disks were glued into the sample carrier using the biocompatible Vulcanizing Adhesive for Spaceflight Experiments (Wacker Chemie AG, certified by ECSS – European Cooperation for Space Standardization). For each experimental group, three quartz disks were used as ground laboratory controls and remained in normal atmospheric conditions at room temperature (22°C) on the bench until analysis (Table 2).

**TABLE 2:** Overview of experimental design.

Condition	Experimental treatment
Lab Control	5-month desiccation (air-dried, 22°C, Earth atmosphere)
Bottom Layer (UV shielded)	5-month desiccation in Mars atmosphere and pressure. Exposure to temperature fluctuation during balloon flight.
Top Layer (UV exposed)	Same as bottom layer with additional exposure to stratospheric UV radiation during balloon flight.

The full mission timeline extended over 5 months including; (i) sample preparation and Trex-Box sample accommodation; (ii) MARSBOx payload integration; (iii) balloon flight; (iv) shipping and sample retrieval from Trex-Box; and (v) sample analysis. During the 5-month experimental duration, both flight and control samples were kept desiccated on quartz disks. Table 2 provides an overview of the conditions microbial samples experienced in this study.

### Estimated UV Radiation Dose

The total UV dose [J m^–2^] that the samples were exposed to during the MARSBOx mission was calculated as follows:

Dose(Jm)-2=[Fluence(mWcm)-2×Time(s)]×10

where UV fluence [mW cm^–2^] values for UVA-UVB (280–400 nm) in the middle stratosphere were taken from a previous flight = 6 mW cm^–2^ (Caro et al., 2019), and sample exposure time [in seconds] = 19140 s. Samples were exposed to an estimated total of 1148 kJ m^–2^ of UVA-UVB radiation. Previously modeled UVC (206–280 nm) values by Caro et al. (2019) ranged from about 0.1–1 μW cm^−2^ for the altitude flown during the MARSBOx mission (∼38 km).

### Ionizing Radiation Dosimetry: M-42

In order to determine the ionizing radiation environment during the flight, a miniaturized, low-power consumption radiation detector system (M-42) was included onboard the MARSBOx payload. The M-42 instrument (size: 142 mm × 38 mm × 13 mm) was developed at DLR and can actively measure the absorbed dose using a silicon detector diode (Berger et al., 2019). Two batteries allow the M-42 to operate as a stand-alone radiation detector system, but for the MARSBOx flight the instrument was externally powered through the in-built micro-USB connector. Upon launch of the balloon, power was provided and the M-42 started taking measurements. Data were stored every 5 min on a non-volatile flash memory and upon landing the instrument was switched off.

### Balloon Flight Profile

One week before flying, the MARSBOx hardware (without biological specimens loaded) was tested in a hypobaric chamber at the Columbia Scientific Balloon Facility (Palestine, TX, United States) to validate system performance and commands. The payload was then transported to the launch site at Ft. Sumner, NM, United States (lat 34.49° long −104.2°), where it was mounted onto the top portion of the LDB #697NT gondola. After installation and prior to launch, the payload surface was sprayed with sterile air and wiped down with isopropyl alcohol. The mission carrying the MARSBOx payload was launched on 23 September 2019 at 1400 UTC, with a full video replay of the flight available here: https://www.youtube.com/watch?v=Vn8qx_0FmV0. The balloon ascended for 2.5 h until reaching an average float altitude of 38.2 km where it remained for 4 h, followed by a 35-min descent on parachute, landing 172 km west of the launch site (lat 35.29° long −105.1°). Sample exposure began during ascent at 21.4 km with the Trex-Box shutter opening at 1521 UTC and concluded 5 h and 19 min later with the Trex-Box shutter closing during descent at 22.0 km at 2040 UTC (Figure 4).

The M-42 dosimeter was turned on at 1405 UTC when the payload was at 3.07 km and remained on until 2119 UTC at 1.75 km above the balloon landing site. Personnel from CSBF recovered the payload on 24 September 2019 and transported it back to the launch site facility inside a climate-controlled vehicle before shipping to NASA KSC at ambient conditions. Three weeks later, samples and instruments (Trex-Box and M-42) were removed from the MARSBOx payload and shipped to the DLR for post-flight analysis.

### Post-flight Processing

After shipment arrival of the samples at the DLR, the Trex-Box was opened within an anaerobic chamber (COY Laboratory products) to ensure a constant low relative humidity (<13% relative humidity). The quartz disks harboring dried cells and spores were retrieved from the carrier and placed inside 2 mL Eppendorf tubes with 1 mL PBS respectively. For *A. niger* spore recovery 2 mm glass beads were added. The tubes were vortexed for 30 s to separate the cells from the disk, and the resulting suspension was used for downstream analyses.

### Determination of Microbial Survival via Standard Plate Counting

The post-flight survival of the tested microorganisms was determined by standard plate counting, where serial dilutions (1:10) were plated on nutrient agar. For bacteria, TSA medium/marine medium was used; for *A. niger* minimal medium supplemented with 0.05% Triton-X was used. Agar plates were incubated for 1–3 days at 37°C for bacteria and 30°C for fungi. Colony forming units (CFU) were counted, and the colony forming units per mL (CFU mL^–1^) were calculated. The survival fraction was calculated as N/N_0_, in which N is the CFU mL^–1^ after sample retrieval and N_0_ is the initial cell concentration on the quartz disk. Determination of CFU mL^–1^ included at least three biological replicates per tested strain (*n* ≥ 3).

### Determination of Metabolic Activity via Resazurin Reduction

To evaluate the potential for revival after exposure to Mars-like conditions, the metabolic activity of the bacterial cells and fungal spores was measured in a 96-well-plate using resazurin reduction as an indicator (alamarBlue^TM^ Cell Viability Reagent, Thermo Fisher^TM^). In each well there was a total volume of 200 μL (130 μL of media, 50 μL of dilutions, and 20 μL of alamarBlue^TM^). The media used was dependent on the microorganism tested and is summarized in Table 1. The plate was incubated for 44 h at 30°C. OD_600_ and OD_570_ were measured every 30 min in a Multi-Detection Microplate Reader (Infinite M200 PRO, Tecan). Orbital shaking of the plate occurred before each measurement. The percentage of reduced resazurin reagent was calculated according to the standard protocol obtained from Thermo Fisher^TM^.

### Determination of *A. niger* Spore Germination

To determine the post-flight germination of *A. niger* spores, spore suspensions (10^6^ spores mL^–1^) from each tested condition were drop plated (3 μL), in triplicate, on MM agar supplemented with 0.003% yeast extract. Plates were incubated at 22°C for 18–27 h. After incubation, light microscopy was used to quantify the number of germinated (G) and non-germinated (NG) spores. At least 200 spores were counted per replicate. Germination was calculated as the average of the G/NG ratio of each replicate per tested condition.

### Evaluation of Spore Cell Wall Integrity

To test spore cell wall integrity after exposure to Mars-like environmental stress, CFUs were quantified for *A. niger* grown in the presence of an antifungal compound that acts on the cell wall (caspofungin) and a cell wall stressor (calcofluor white). Spore suspensions from the three exposure conditions (desiccated lab control, UV shielded bottom layer, and UV exposed top layer) and fresh spores (as non-desiccated control) were serially diluted in a 96-well plate. For each dilution (10^–1^ to 10^–5^), 5 μL were spotted on MM nutrient agar supplemented with 0.75 μg mL^–1^ caspofungin diacetate (Sigma) or 40 μg mL^–1^ calcofluor white (Sigma), and incubated for 2–4 days at 30°C.

### Statistical Analysis

Survival, metabolic activity and germination data were plotted as mean values using SigmaPlot (Version 13.0, Systat Software). Error bars are presented as standard error (SE). Student’s *t*-test and the non-parametric Mann–Whitney test were performed with Mean + SE to identify significant differences between each two tested conditions, per microorganism. A two-tailed *p*-value of *p* ≤ 0.05 was considered significant. ANOVA analysis was also performed on survival data. A summary of *t*-test and ANOVA analysis of survival data can be found in Supplementary Tables 1–3.

## Results

### Middle Stratosphere as a Mars Analog Environment

This study exposed different microorganisms (*Aspergillus niger*, *Staphylococcus capitis* subsp. *capitis, Salinisphaera shabanensis*, and *Buttiauxella* sp. MASE-IM-9) to a Mars analog environment. The robust simulation of Mars environmental conditions was made possible with access to Earth’s middle stratosphere onboard a scientific balloon flight, where combined conditions include elevated non-ionizing and ionizing radiation doses, low temperature, and extreme desiccation. Additionally, samples were flown inside a Trex-Box container with Mars gas composition (mostly CO_2_) and surface atmospheric pressure (5–10 mbar). Onboard the MARSBOx payload, microbial samples were exposed as dried cells or spores desiccated on quartz disks in two different layers: a bottom layer that was shielded from UV radiation, and a top layer that was exposed to stratospheric UV conditions. Table 3 summarizes the environmental conditions in the balloon flight compared to the generalized equatorial surface of Mars based on available measurements and models.

**TABLE 3:** Environmental conditions aboard balloon flight compared to martian conditions.

Parameters	Balloon Flight + Trex-Box (7–38 km alt.)	Mars (at equator)
UV fluence (280 – 400 nm)	∼6 mW cm^–2*a*^	∼5 mW/cm^–2*b*^
Temperature (min.)	–51°C	–73°C
Temperature (max.)	+21°C	+20°C
Sample exposure duration	5 h 19 min	–
Atmosphere composition	95% CO_2_	96% CO_2_
	1.9% Ar	1.9% Ar
	2.6% N_2_	1.9% N_2_
	0.17% O_2_	0.14% O_2_
	0.07% CO^*c*^	0.07% CO^*d*^
Atmospheric pressure	5–10 mbar	5–10 mbar^*e*^
Total UV dose (est.)	∼1148 kJ m^–2*f*^	-

*^*a*^Fluence rates for stratospheric UVA-UVB were measured in a previous balloon flight (Caro et al., 2019).*

*^*b*^Schuerger et al., 2003.*

*^*c*^Mars gas in Trex-Box was ordered from Boggs Gases, Inc. (Titusville, FL, United States) as a commercial mixture of the top five gasses in the martian atmosphere (Schuerger et al., 2008).*

*^*d*^Mahaffy et al., 2013.*

*^*e*^Barlow, 2008.^*f*^Total estimated UV dose that samples were exposed to during the balloon flight (see section “Estimated UV radiation dose”).*

### M-42 Ionizing Radiation Data

**FIGURE 5:**
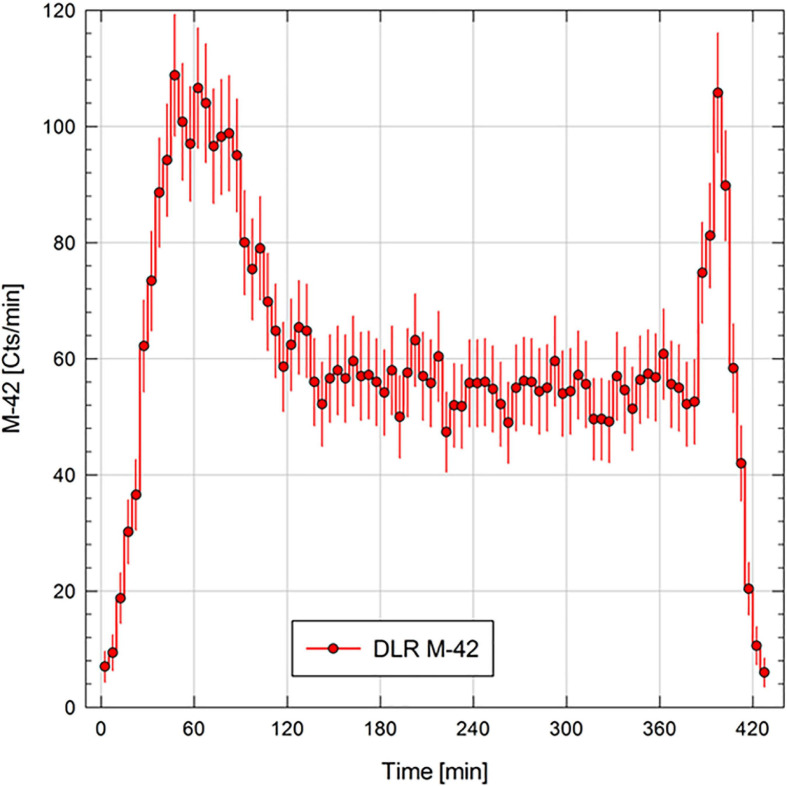
The M-42 count rate (cts min^–1^) measured for the MARSBOx balloon flight mission.

In the following sub-section, we will only provide a snapshot of the data measured with the M-42 instrument to demonstrate proof of operations during the mission. In Figure 5, we provide the count rate of the silicon diode for the whole time the system was powered. The count rate plot shows the crossings of the Regener maximum (Regener and Pfotzer, 1935), during ascent and descent of the balloon. At cruising altitude, we saw a nearly constant count rate which results in a dose rate of 75.5 ± 13 μGy per day. In total we measured a dose of 20.9 μGy for the whole mission, which is equivalent to around 10 days of natural background radiation received in the DLR laboratory in Cologne.

### Stratospheric Sunlight Reduced Microbial Survival

**FIGURE 6:**
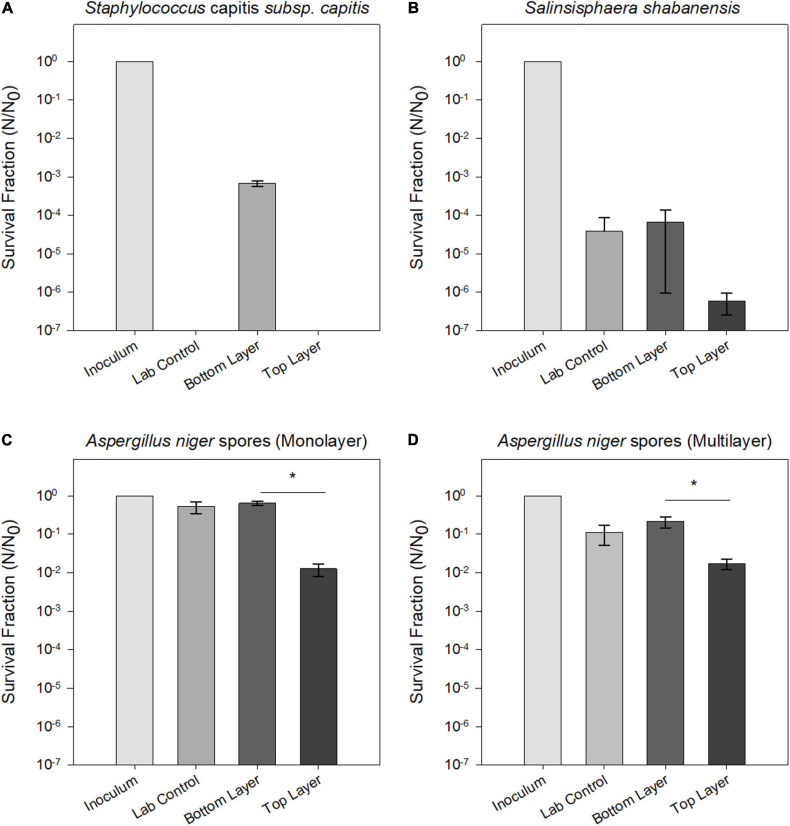
Survival fraction (N/N0) of tested strains after exposure to Mars simulated conditions aboard the MARSBOx payload. **(A)** *S. shabanensis* **(B)** *S. capitis* subsp. *capitis*. **(C,D)** *A. niger* spores in monolayer **(C)** and in multilayer **(D)**. Data for *Buttiauxella* sp. MASE-IM-9 are not shown, since no surviving cells were recovered after exposure.

Figure 6 summarizes survival fractions for exposed microorganisms in the balloon experiment. Spores from the filamentous fungus *A. niger* showed the highest endurance to the combined stresses of stratospheric radiation and simulated martian atmospheric and temperature conditions during the MARSBOx flight (Figure 6D). The extremophilic bacterium *S. shabanensis* tolerated desiccation but showed a decrease in survival in the UV-exposed layer of the Trex-Box (Figure 6B). The human-associated *S. capitis* subsp. *capitis* also displayed sensitivity to UV exposure, with only cells from the UV-shielded bottom layer surviving the flight experiment (i.e., still exposed to Mars gas, desiccation and temperature fluctuation). It should be noted that the laboratory control cells of *S. capitis* subsp. *capitis*, kept desiccated under oxic conditions, were not revivable. *Buttiauxella* sp. MASE-IM-9 showed no growth, even in the laboratory controls; consequently, stratosphere exposure effects could not be determined for this microorganism.

### Survival and Metabolic Activity of *Staphylococcus capitis* subsp. *capitis*

**FIGURE 7:**
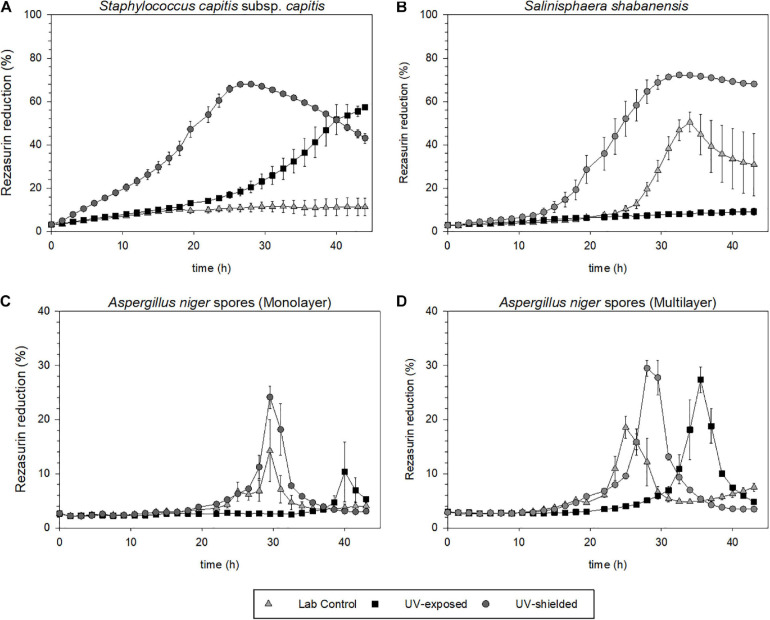
Metabolic activity upon revival, after exposure to Mars simulated conditions aboard the MARSBOx payload. Metabolic activity is depicted as percentage of reduced resazurin. **(A)** *S. shabanensis* **(B)** *S. capitis* subsp. *capitis*. **(C,D)** *A. niger* spores in monolayer **(C)** and in multilayer **(D)**.

For *S. capitis* subsp. *capitis*, the average of three samples (*n* = 3) from flight conditions and six samples (*n* = 6) from the laboratory control are shown in Figure 6A. In the UV-exposed samples and the laboratory controls, no surviving cells could be detected via determination of CFU mL^–1^. The UV-shielded samples showed a significant reduction of the survival fraction by three orders of magnitude (*p* = 0.03). Metabolic activity was detected in UV-shielded (bottom layer) and UV-exposed (top layer) samples, but not in the laboratory controls. Metabolic activity in the UV-exposed cells was delayed in comparison to UV-shielded cells (Figure 7A). UV-shielded cells reached the maximum reduction of resazurin (70%) after 28 h of incubation, whereas in UV-exposed cells, the resazurin reduction was still below 70% after 44 h (total incubation time).

### Survival and Metabolic Activity of *Buttiauxella* sp. MASE-IM-9

For *Buttiauxella* sp. MASE-IM-9, the survivability of cells dried in medium or buffer was evaluated. This strain did not survive laboratory controls or the MARSBOx flight samples. Similarly, with resazurin assay no metabolic activity was detected for *Buttiauxella* sp. MASE-IM-9, in any of the samples.

### Survival and Metabolic Activity of *Salinisphaera shabanensis*

No survival differences between *S. shabanensis* cells dried in medium and *S. shabanensis* cells dried in buffer were observed. Therefore, the average of six samples (*n* = 6) was reported in Figure 6. While *S. shabanensis* was able to endure 5 months of desiccation, there was still an overall reduction of four orders of magnitude (laboratory control, Figure 6B). The survival for the laboratory control and the UV-shielded cells was similar (*p* = 0.725), with UV exposure further reducing the survival fraction (*p* = 0.602) (Figure 6B). These results were supported by the metabolic activity assay with resazurin reduction in both laboratory controls and flown UV-shielded cells (Figure 7B).

### Survival and Metabolic Activity of *Aspergillus niger* Spores

Compared to other microorganisms evaluated in our experiment, *A. niger* spores were the most resistant to all tested conditions (Figure 6). Two different *A. niger* spore concentrations were tested in the Trex-Box: 10^7^ spores mL^–1^ (spore monolayer) and 10^8^ spores mL^–1^ (spore multilayer); *n* = 3 for each concentration (Figure 1). Survival of UV-exposed spores was reduced by two orders of magnitude compared to laboratory controls, in both spore monolayer (*p* = 0.001) and spore multilayer (*p* = 0.001). Survival of UV-shielded spores, i.e., still exposed to Mars gas, pressure and temperature, was not affected, when compared to laboratory controls, in either the monolayer (*p* = 0.592) or multilayer (*p* = 0.495) concentration (Figures 6C,D, respectively). When assessing the time taken to reach the maximum of metabolic activity, UV-exposed monolayer spores were delayed by 48% (peaking only after 43 h of incubation) when compared with laboratory control (peaking after 29 h of incubation); and multilayer spores were delayed by 38% (peaking only after 36 h of incubation) when compared with laboratory control (peaking after 26 h of incubation) as shown in Figures 7C,D, respectively.

**FIGURE 8:**
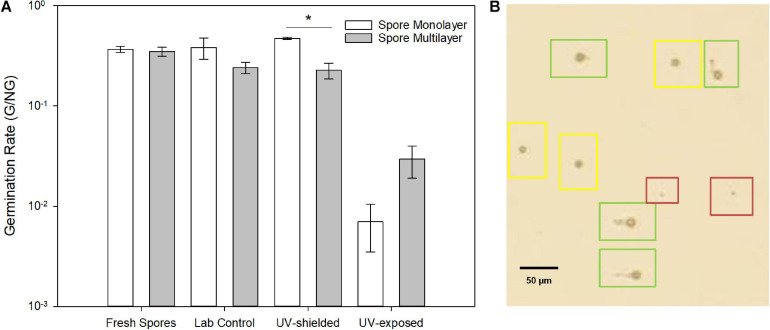
**(A)** Germination rate (G/NG) of *A. niger* spores after exposure to simulated Mars conditions. **(B)** Light microscopy showing resting spores (red), swollen spores (yellow) and germinated spores (green). Both resting and swollen spores were counted as ungerminated.

**FIGURE 9:**
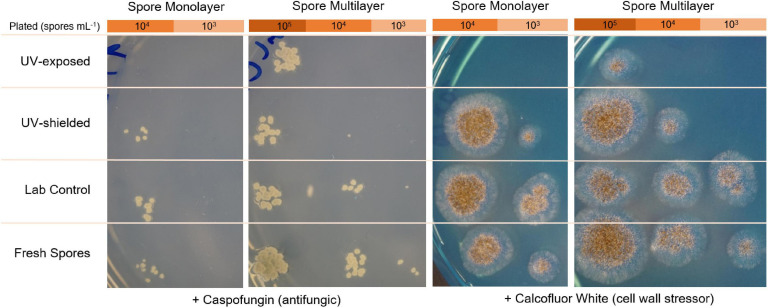
Stress resistance of *A. niger* towards caspofungin (antifungal compound) and to calcofluor white (cell wall stressor), after spore exposure to simulated martian conditions.

Spore germination was delayed by 22% in UV-exposed spores, being detected only after 27 h, versus 22 h of laboratory control. Germination rate was significantly lower in UV-exposed when compared to UV-shielded spores (*p* = 0.01 monolayer; *p* < 0.001 multilayer) and when compared to laboratory controls (*p* = 0.03 monolayer; *p* = 0.08 multilayer) (Figure 8). UV-shielded spores showed decreased ability to cope with cell wall stress (1 order of magnitude; whereas UV-exposed spores were shown to be highly sensitive to cell wall stress, 2 or more orders of magnitude) (Figure 9).

Survival, germination, and metabolic activity were compared between spore monolayer and spore multilayer (Table 4). In UV-shielded martian-like conditions, the presence of a multilayer was shown to significantly decrease survival (*p* = 0.01) (Figure 6) and germination rate (*p* = 0.004) (Figure 8), but to significantly increase metabolic activity (*p* = 0.02) (Figure 7). In UV-exposed, Mars-like conditions, the presence of a spore multilayer did not affect survival (*p* = 0.5) or germination rate (*p* = 0.1); but was shown to increase metabolic activity (*p* = 0.04). Multilayer spores were significantly faster in reaching the metabolic maximum than monolayer spores, in all tested conditions (Table 4).

**TABLE 4:** Impact of spore monolayer versus spore multilayer in *A. niger* resistance to Mars-like conditions.

		Monolayer	Multilayer	^*f*^	*p*-value
***Survival fraction ^*a*^***	**Lab Control**	5.3 × 10^–1^ ± 1.8 × 10^–1^	1.1 × 10^–1^ ± 5.9 × 10^–2^	↓	0.09
	**UV shielded**	6.4 × 10^–1^ ± 8.0 × 10^–2^	2.1 × 10^–1^ ± 6.6 × 10^–2^	↓	0.01*
	**UV exposed**	1.3 × 10^–2^ ± 4.5 × 10^–3^	2.1 × 10^–1^ ± 5.2 × 10^–3^	↑	0.5
***Metabolic max. ^*b*^***	**Lab Control**	20% ± 5	23% ± 1	↑	0.6
	**UV shielded**	29% ± 1	36% ± 0.1	↑	0.002*
	**UV exposed**	13% ± 7	35% ± 0.5	↑	0.04*
***Time until max. ^*c*^***	**Lab Control**	29 h ± 1.3	26 h ± 0.7	↓	0.1
	**UV shielded**	30 h ± 0.3	29 h ± 0.3	↓	0.07
	**UV exposed**	44 h ± 2	36 h ± 0.6	↓	0.002 *
***Germination rate^*d*^***	**Lab Control**	0.38 ± 0.09	0.24 ± 0.03	↓	0.2
	**UV shielded**	0.47 ± 0.01	0.23 ± 0.04	↓	0.004 *
	**UV exposed**	0.01 ± 0.00	0.03 ± 0.01	↓	0.1

*^*a*^Survival fraction calculated as N/N_0_.*

*^*b*^Metabolic activity maximum, measured as % of reduced resazurin.*

*^*c*^Incubation time taken to reach maximum of metabolic activity (h).*

*^*d*^Time of incubation until metabolic peak was detected (h).*

*^*f*^Presence of spore multilayer has increased (↑) or decreased (↓) the measured parameter.*

## Discussion

### Relevance to Space Biology and Astrobiology

In this study, bacterial and fungal strains were exposed to Mars-like environmental conditions onboard the MARSBOx payload during a 7 h balloon flight to the middle stratosphere (∼38 km). The tested microorganisms (*Aspergillus niger*, *Staphylococcus capitis* subsp. *capitis, Salinisphaera shabanensis*, and *Buttiauxella* sp. MASE-IM-9) were chosen based on astrobiology and space biology relevance. Moreover, the choice of these strains was to provide a representative set of samples that are capable of demonstrating the MARSBOx experimental design as a valuable martian analog for future investigations.

To test the hypothesis that isolates from extreme Mars-analog environments on Earth would be able to survive the MARSBOx simulated martian conditions, two extremophilic bacteria were included. The bacterium *S. shabanensis* is a halophile isolated from the brine–seawater interface of the Shaban Deep at a depth of 1.3 km where the maximal salinity reaches 26% (Eder et al., 2002). Deep-sea brine pools have been identified as martian analogs in regards to the salinity and water activity in brines (Antunes, 2020). On the surface of Mars, brines might remain liquid at ultra-low temperatures (Toner and Catling, 2016). In addition to high salinity, the martian surface lacks oxygen and nutrients. For these reasons, the Gram-(-) bacterium, *Buttiauxella* sp. MASE-IM-9, isolated from an anoxic, nutrient-limited, and sulphidic martian analog spring in Germany (Cockell et al., 2018), was also included in our study.

An emerging body of evidence shows that spores from the fungus *A. niger* can withstand harsh conditions (e.g., radiation, heat, low water activity, etc.) (Singaravelan et al., 2008; Esbelin et al., 2013; Segers et al., 2018; Cortesão et al., 2020a), probably due to the roles of pigmentation, cell wall structure, and metabolic suppression, which might enable them to survive space travel on the outer surfaces of a spacecraft, and to thrive within the spacecraft’s controlled habitat. Alarmingly, the inhalation of *A. niger* spores may cause respiratory diseases, especially when in closed indoor habitats such as the ISS, which facilitate spore dispersal (Silverman et al., 1967; Latge, 1999; Esbelin et al., 2013; Cortesão et al., 2020a, b). This motivates further study on how the species responds to spaceflight conditions, and consequent implications for astronaut health, in particular in long-term space missions. Moreover, on Earth, *A. niger* is used in biotechnology to produce a wide-range of useful compounds including citric acid, antibiotics, and enzymes (Meyer et al., 2011; Cairns et al., 2018). Consequently, *A. niger* could play an important role in human space exploration as long-duration, far-reaching, missions may require biomanufacturing and resource-independence from Earth (Silverman et al., 1967; Latge, 1999; Esbelin et al., 2013; Cortesão et al., 2020a, b).

Finally, *S. capitis* is a Gram-(+) bacterium commonly associated as a commensal species on human skin (Byrd et al., 2018). However, *S. capitis* also has the ability to cause infections in neonates and form biofilms on implants (de Silva et al., 2002; Cui et al., 2013). The *Staphylococcus capitis* subsp. *capitis* strain K1-2-2-23 (DSM 111179) used in this study was isolated aboard the ISS within an indoor exposure experiment (Sobisch et al., 2019). Its occurrence in crewed space stations, the clinical relevance and the phylogenetic proximity to other clinically relevant staphylococcal species make *S. capitis* a useful model organism to study the effects of space conditions on opportunistic human pathogens, and to identify potential risks of crew infection (Xiao et al., 2019).

### Radiation Levels in the Middle Stratosphere

Life on Earth is protected from low-wavelength UV radiation (100–280 nm) by atmospheric ozone (Horneck et al., 2010). Above the concentration of atmospheric ozone, where large scientific balloon missions float, UV radiation levels nearly match those expected on the surface of equatorial Mars. For instance, Caro et al. (2019) recently measured an average instantaneous UVA-UVB flux of ∼6 mW cm^−2^ on a meteorological balloon mission flown to the middle stratosphere; the total combined dose measured was 1.9 kJ. In comparison, ∼5 mW cm^–2^ was the reported value to be expected at the surface of Mars for UVA-UVB according to calculations from Schuerger et al. (2003). Surprisingly few UVC measurements have been obtained for Earth’s middle stratosphere but Caro et al. (2019) modeled an expected range of 0.1–1 μW cm^–2^ for altitudes above the ozone layer. Besides intense UV radiation (derived levels in this study), we measured ionizing radiation in the middle stratosphere with the M-42 active dosimeter. This was the first successful M-42 flight test in preparation for the dosimetry suite onboard the upcoming Matroshka AstroRad Radiation Experiment on the NASA Artemis I mission (Berger et al., 2019). At the ∼38 km float altitude on the MARSBOx mission, the dose rate was almost constant, around 75.5 ± 13 μGy day^–1^. In comparison, the dose rate measured on the surface of Mars for the same time period would approach 260 μGy day^–1^. A more comprehensive radiation biophysics analysis (e.g., Monte Carlo calculations) will be reported later alongside additional balloon flight measurements from a joint NASA-DLR long-duration Antarctic mission flown in December 2019. In the meantime, we point readers interested in the ionizing radiation levels of the middle stratosphere over New Mexico to the RaD-X mission results from Mertens (2016).

### Bacterial Survival

The endurance of *S. shabanensis*, a non-pigmented halophile, to the (derived) UV dose of 1148 kJ m^–2^ during the balloon flight was unanticipated considering the original isolation source for the species was a deep-sea brine pool with no direct illumination from sunlight. Our stratosphere balloon mission results for *S. shabanensis* (partial resistance to UV exposure) reveal a wide variability in UV response based on the model bacterium studied. It was previously reported that even vegetative *Escherichia coli* cells can persist for 7 days under simulated Mars conditions, even when exposed to 8 h of UVC irradiation (200–280 nm) at a fluence of 3.6 W m^–2^ per day (Berry et al., 2010). Besides innate physiological differences, survival rates might also be influenced by the degree to which the UV dose was attenuated. With past laboratory- and flight-based experiments embedding microorganisms in different substrates – including Mars analog soils – the effects of UV (alone) can be difficult to assess (Rettberg et al., 2004; Wadsworth and Cockell, 2017). Cell layering (discussed later in section “*A. niger* spores survive Mars-like conditions”) also likely plays a role in variable survival outcomes, as reported with past stratosphere exposure studies (Khodadad et al., 2017). We expected *S. capitis* subsp. *capitis* to be more tolerant to radiation in the stratosphere because of its natural occurrence on human skin (Byrd et al., 2018), where direct illumination from sunlight would be common. For instance, the average UV dose causing erythema (abnormal redness of the skin) of Americans is approximately 25 kJ m^–2^ per year (Godar et al., 2001).

Whether the tested bacterial strains would be able to survive in a real Mars-surface environment depends on various aspects; however, access to UV-shielding will certainly play a major role. This was seen in our study, as both the halophilic bacterium *S. shabanensis*, and the human skin associated bacterium *S. capitis* subsp. *capitis* survived the UV-shielded Mars-like environment during the balloon flight. In the event that these bacteria are brought to Mars, either in robotic missions for astrobiological research purposes; or by accident through crew-led contamination in space missions (Avila-Herrera et al., 2020) our results suggest that bacterial bioburden embedded deep inside of spacecraft sent to Mars might remain viable for longer periods of time (>5 h).

### Bacterial Desiccation Tolerance in Mars-Like Conditions

Desiccation can be a stressful condition for cells, where the accumulation of reactive oxygen species and irreversible changes in lipids, proteins, and nucleic acids can lead to death (Cox, 1993; Dose and Gill, 1995). Some microorganisms can tolerate extreme desiccation by ceasing metabolic activity in a state of anhydrobiosis (Glasheen and Hand, 1988; Wright, 1989; Potts, 1994). Our results for the bacterial species *S. shabanensis* showed survival under long-term desiccation and sunlight exposed flight conditions. The survival for the laboratory control and the UV-shielded organisms are similar, which may indicate a desiccation sensitivity of this strain. Cells of *S. capitis* subsp. *capitis* K1-2-2-23 were inactivated from the flight UV-exposure but did partially survive the UV-shielded layer of the Trex-Box. Interestingly, the desiccated laboratory controls for *S. capitis* subsp. *capitis* did not survive the experiment, indicating that long-term resistance to desiccation was only possible when cells were under the Mars-like atmosphere and not when kept in Earth atmosphere. The results from the balloon flight warrant further investigation to determine if a Mars gas mixture has an impact on the stability of some bacterial species. For instance, it is known that the presence of oxygen can decrease the survivability of prokaryotes during desiccation (Potts, 1994; Vriezen et al., 2007; Beblo-Vranesevic et al., 2017b) and in additional experiments we observed that the survival of *S. capitis* subsp. *capitis* K1-2-2-23 increased when desiccation occurred in anoxic conditions (data not shown). Finally, the last bacterial species flown in this experiment, *Buttiauxella* sp. MASE-IM-9, did not survive long-term desiccation. Therefore, no surviving cells could be detected in the balloon flight samples or from laboratory controls. This negative result was consistent with previous Mars analog experiments where *Buttiauxella* sp. MASE-IM-9, a facultative anaerobe, survived a maximum of 3 months of desiccation (Beblo-Vranesevic et al., 2018, 2020).

### *A. niger* Spores Survive Mars-Like Conditions

While bacterial cells were sensitive to UV exposure in the middle stratosphere, in addition to long-term desiccation, *A. niger* spores were highly resistant to all tested conditions. Laboratory controls demonstrated *A. niger* spore endurance to 5-month desiccation, when air-dried and kept at room temperature (22°C) at the bench, under Earth atmospheric conditions. Spores of *A. niger* shielded from UV (bottom layer) endured a 5-month desiccation within Mars-like atmosphere and pressure, with additional exposure to extreme temperature fluctuations during the balloon flight (−51°C to +21°C). Finally, spores of *A. niger* exposed to UV (top layer) withstood over 5 h of full simulation of Mars environmental conditions, i.e., exposed to Mars gas, atmosphere, temperature fluctuation, and to a total estimated UVA-UVB dose of 1148 kJ m^–2^ during the balloon flight. A previous study testing *A. niger* spore survival to desiccation and solar radiation was done in an experimental setting similar to this study: *A. niger* spore monolayers were dried in glass disks and exposed to ∼16 kJ m^–2^ of UVB (280–320 nm); the specimens were highly resistant, with 24% ± 5% survival (Dose et al., 2001). The extraordinarily high level of resistance of *A. niger* spores to UVC radiation (LD_90_ value of 1038 J m^–2^) has been previously reported by Cortesão et al. (2020a). Another balloon-flown study (i.e., same launch site and season; different year) with pigmented spores of the fungus *Fuligo spectica* showed that these remained viable after a 9 h exposure to conditions in the stratosphere (Díez et al., 2020). In the *Aspergillus* genus, secondary metabolites e.g., DHN-melanin (a pigment) and fumiquinazoline might be associated with UV protection roles (Blachowicz et al., 2020).

The ubiquitous presense of *A. niger* spores in human indoor-closed habitats, and their high resistance to outer space conditions, suggests these will likely travel with us to Mars. A contamination scenario, several factors might affect the survivability potential of *A. niger* spores in a Mars-surface environment. Some are intrinsic to the spores, e.g., molecular mechanisms such as DNA repair systems; or structural protection mechanisms such as the thick cell wall (Latgé et al., 2017). Whereas other factors are external, for instance, shielding from the spacecraft surfaces or martian regolith. An important factor known to impact survival outcomes is the starting cell concentration (Khodadad et al., 2017). To test this, we compared *A. niger* responses to the stratosphere as either a spore monolayer (10^7^ spores ml^–1^) or a spore multilayer (10^8^ spores ml^–1^) (Table 4). When exposed to UV-shielded conditions, a higher starting spore concentration (multilayer) influenced all primary measures (i.e., survival fraction, spore germination, and metabolic activity). Unexpectedly, spores in a multilayer yielded a decreased survival in laboratory controls and UV shielded conditions, as well as a decreased germination rate in all tested conditions, when compared with spores in a monolayer. These discrepancies might simply be due to incomplete removal of biomass from the quartz disks or due to the presence of hyphae fusion in germination test plates; either of which could lead to undercounting.

Moreover, in *A. niger* spores, the cell wall is a highly complex structure that plays an important role in protecting the spores from extracellular environmental stress. The spore cell wall is composed mainly of polysaccharides (α-glucans β-glucans), galactomannan, and chitin; and is surrounded by a rodlet layer with hydrophobic surface proteins, and a melanin layer. When germinating into vegetative cells (hyphae), the spore cell wall is remodeled and no longer provides protection to extreme conditions (Latgé et al., 2017). Considering environmental changes typically act first on the cell wall, we evaluated how Mars-like conditions might alter *A. niger* spore cell wall integrity. All tested spores were able to revive (i.e., germinate) and grow in media supplemented with cell wall stressors: calcofluor white or caspofungin. Calcofluor white is a non-specific fluorochrom that can bind to 1,3- and 1,4-β polysaccharides on chitin and cellulose, inhibiting chitin microfibril assembly and cell wall integrity (Fiedler et al., 2014).

## Conclusion

To date, a variety of terrestrial analogs and simulation chambers have been used to predict outcomes for microbial exposure to Mars-like conditions. Most often, such investigations use elevated radiation and desiccation paired with low pressure and temperature alongside a Mars gas mixture (Jensen et al., 2008; Schuerger et al., 2008; Motamedi et al., 2015). However, few studies can simultaneously recreate a multi-factor Mars environment. The renewed focus on Mars robotic and human exploration e.g., Mars 2020, Mars Sample Return (NASA, 2020) and ExoMars 2022 (ESA, 2020) amplifies the need for additional Mars analog studies in the coming years. In this study we:

• reported the use of a new scientific payload (MARSBOx) for stratospheric balloon missions allowing access to a wide-ranging Mars analog environment with natural ionizing and non-ionizing radiation;
• demonstrated a successful experimental set-up, of the Trex-Box and MARSBOx combined, where four different microorganisms could be tested, in dried conditions, throughout a 5-month period, without cross-contaminations.
• showed that the extremophilic bacterium *S. shabanensis* and the human skin-associated bacterium *S. capitis* subsp. *capitis* survived the UV-shielded Mars-like environment during the balloon flight, suggesting that bioburden embedded deep inside of spacecraft sent to Mars might remain viable for longer periods of time;
• revealed that highly pigmented spores from the fungus *A. niger* would survive, in a Mars-like middle stratosphere environment for > 5 h of UV exposure, even as a spore monolayer (10^6^ spores mL^−1^), i.e., with no self-shielding.

Taken together, we conclude pigmented fungal spores might be considered some of the most likely forward contaminants to survive if inadvertently delivered to Mars. Our results underscore the importance of including fungal spores in Mars forward contamination studies and relevant planetary protection policies, which currently restrict surface bioburden of ≤3 × 10^5^ bacterial endospores for robotic lander systems that are not carrying instruments to investigate extant martian life (category IVa) (COSPAR, 2020). Moreover, fungal spore sensitivity to extreme heat or to high doses of combined sources of space radiation, as well as to other factors that affect survivability (e.g., regolith reactive compounds or regolith shielding), should be further evaluated to better assess the forward contamination potential in Mars analog environments.

## Data Availability Statement

The original contributions presented in the study are included in the article/Supplementary Material, further inquiries can be directed to the corresponding author/s.

## Author Contributions

MC, KS, SK, and KB-V performed the microbial experiments, analyzed the data, and wrote the manuscript. DS, SW, SV, ER, and RM contributed to the conception and design of the study, data analyses and manuscript preparation. DS, ML, LJ, and PJ prepared and performed the balloon flight mission and contributed to manuscript preparation. TB contributed with the M-42 experiment and manuscript preparation. All authors contributed to the article and approved the submitted version.

## Conflict of Interest

The authors declare that the research was conducted in the absence of any commercial or financial relationships that could be construed as a potential conflict of interest.

## Supplementary Material

Supplementary Figure 1 Environmental data from the flight via CSBF (outside temperature and pressure and altitude).

Supplementary Table 1 Student’s *t*-test was used to identify significant differences between two tested conditions of the survival data using raw data (CFU/ml). Two-tailed *p*-value is presented. Equal variances assumed.

Supplementary Table 2 Student’s *t*-test was used to identify significant differences between two tested conditions of the survival data (Survival Fraction N/N_0_) using mean values and standard error. Two-tailed *p*-value is presented. Equal variances assumed.

Supplementary Table 3 ANOVA on Ranks followed by Kruskal-Wallis and Dunn’s test were used to identify significant differences in the survival data (CFU/ml).

Supplementary Video 1 Rotatable shutter opening to start sample exposure to UV radiation, once stratosphere altitudes were reached.

Supplementary Video 2 Rotatable shutter closing to stop sample exposure to UV radiation, in preparation for descent.
