# Supplementary material for: MARSBOx: Fungal and Bacterial Endurance From a Balloon-Flown Analog Mission in the Stratosphere
Source: Front Microbiol. 2021 Feb 22;12:601713. doi: 10.3389/fmicb.2021.601713 (PMC7937622; doi:10.3389/fmicb.2021.601713)
Supplement: Supplementary Figure 1 — Environmental data from the flight via CSBF (outside temperature and pressure and altitude). [file Data_Sheet_1.docx]

Supplementary Material

# Supplementary Figures

## Supplementary Figure 1. Environmental data from the flight via CSBF (outside temperature and pressure and altitude).

# Supplementary Tables

**Supplemental Table 1.** Student’s t-test was used to identify significant differences between two tested conditions of the survival data using raw data (CFU/mL). Two-tailed p-value is presented. Equal variances assumed.

| t-test  Raw data | *Staphylococcus capitis* subsp. *capitis* | *Salinsisphaera shabanensis* | *Aspergillus niger* (monolayer) | *Aspergillus niger* (multilayer) |
| --- | --- | --- | --- | --- |
| Control vs.  UV-shielded | 0.006 * | 0.240 ^a^ | 0.592 | 0.229 ^a^ |
| Control vs.  UV-exposed | 1.000 ^a^ | 0.223 | 0.012^a^ * | 0.016^a^ * |
| UV-shielded vs. UV-exposed | 0.006 * | 0.136 | 0.012^a^ * | 0.012^a^ * |

(^a^) Mann-Whitney test was used for non-Gaussian distributed data.

(^*^) p < 0.05 was considered significant

**Supplemental Table 2.** Student’s t-test was used to identify significant differences between two tested conditions of the survival data (Survival Fraction N/N0) using mean values and standard error. Two-tailed p-value is presented. Equal variances assumed.

| t-test  Mean + Standard Error | *Staphylococcus capitis* subsp. *capitis* | *Salinsisphaera shabanensis* | *Aspergillus niger* (monolayer) | *Aspergillus niger*  (multilayer) |
| --- | --- | --- | --- | --- |
| Control vs.  UV-shielded | 0.003^a^ * | 0.725 | 0.592 | 0.495 |
| Control vs.  UV-exposed | 1.000 | 0.602 | 0.001^a^ * | 0.053 * |
| UV-shielded vs. UV-exposed | 0.003^a^ * | 0.513 | 0.001^a^ * | 0.001^a^ * |

(^a^) Mann-Whitney test was used for non-Gaussian distributed data.

(^*^) p < 0.05 was considered significant

**Supplemental Table 3.** ANOVA on Ranks followed by Kruskal-Wallis and Dunn’s test were used to identify significant differences in the survival data (CFU/mL).

| ANOVA on Ranks followed by Kruskal-Wallis and Dunn’s test | *Staphylococcus capitis* subsp. *capitis* | *Salinsisphaera shabanensis* | *Aspergillus niger*  (monolayer) | *Aspergillus niger* (multilayer) |
| --- | --- | --- | --- | --- |
| Control vs.  UV-shielded | n.a. | 0.905 | 1,000 | 1.000 |
| Control vs.  UV-exposed | n.a. | 0.154 | 0.047* | 0.107 |
| UV-shielded vs.  UV-exposed | n.a. | 0.016* | 0.034* | 0.014* |

(^*^) p < 0.05 was considered significant

n.a. = not applicable, given Do Not Test (DNT) result of analysis; assumes no significant difference.

# Supplementary Videos

**Supplementary Video 1.** Rotatable shutter opening to start sample exposure to UV radiation, once stratosphere altitudes were reached.

**Supplementary Video 2.** Rotatable shutter closing to stop sample exposure to UV radiation, in preparation for descent.

Our two Supplemental Videos can be accessed through here:

- Supplemental Video 1: <https://tubcloud.tu-berlin.de/s/ERLd2dK3xrFSXSj>

- Supplemental Video 2: <https://tubcloud.tu-berlin.de/s/zSiMadtQLxaLyLr>
